# Peripheral neuropathy in patients with human immunodeficiency viral infection at a tertiary hospital in Ghana

**DOI:** 10.1007/s13365-019-00743-0

**Published:** 2019-04-26

**Authors:** Peter Puplampu, Vincent Ganu, Ernest Kenu, William Kudzi, Patrick Adjei, Leticia Grize, Michael Käser

**Affiliations:** 1 Department of Medicine, School of Medicine and Dentistry, College of Health Sciences, University of Ghana, Accra, Ghana; 2 Department of Medicine, Korle-Bu Teaching Hospital, Accra, Ghana; 3 Department of Epidemiology, School of Public Health, University of Ghana, Accra, Ghana; 4 Centre for Tropical Clinical Pharmacology and Therapeutics, School of Medicine and Dentistry, College of Health Sciences, Accra, Ghana; 5 Swiss Tropical and Public Health Institute, Socinstr. 57, 4002 Basel, Switzerland; 6 University of Basel, Petersplatz 1, 4003 Basel, Switzerland

**Keywords:** Peripheral neuropathy, PN, Human immunodeficiency virus, HIV-AIDS, Prevalence, Side effect, Antiretroviral therapy, ART, People living with HIV/AIDS, PLHIV, Biothesiometer, NRTI, Protease inhibitor, Sensory neuropathies

## Abstract

Peripheral neuropathy (PN) is the most frequent neurological complication in people living with HIV/AIDS. Neurological damage was identified to not only be caused by the viral infection itself but also through neurotoxic antiretroviral therapy (ART). PN is associated with a variety of risk factors; however, detailed knowledge is scarce for sub-Saharan African populations, bearing among the highest HIV/AIDS infection burden.

In a cross-sectional study, we assessed the prevalence of PN in 525 adult outpatients suffering from HIV/AIDS and admitted to the largest tertiary hospital in Ghana. Through a detailed questionnaire and clinical examination including neurologic assessment and laboratory blood sample testing, this study investigated associations of PN with demographic and health determinants and identified risk factors associated with sensory neuropathy.

The prevalence of PN in the Ghanaian cohort was 17.7% and increased odd ratios (OR) when patients were taller (> 1.57 m; OR = 3.84; 95% CI 1.38–10.66) or reached the age > 34 years (*p* = 0.124). Respondents with longer education duration had significantly less PN (≥ 9 years of education; OR = 0.49; 95% CI 0.26–0.92). The study also identified significant association of PN to both waist and hip girth and neutrophil counts. Curiously, higher adjusted odd ratios (aOR) of PN of patients under ART treatment were observed when CD4 lymphocytes were elevated (aOR = 0.81; 95% CI 0.36–1.83 and aOR = 2.17; 95% CI 0.93–5.05, for 300 and 600 counts, respectively). For patients on ART, an increase of 10 CD4 cell count units increased their chance of developing PN by 1% (aOR = 1.01; 95% CI 1.00 to 1.03).

Despite current drug application regulations, prevalence of PN is still unacceptably high in sub-Saharan African populations. Reduction in chronic morbidity through a health system with routine monitoring, early diagnosis and prompt intervention, and effective case management can improve people living with HIV/AIDS’ quality of life.

**Electronic supplementary material:**

The online version of this article (10.1007/s13365-019-00743-0) contains supplementary material, which is available to authorized users.

## Introduction

Peripheral neuropathy (PN) in its diverse forms has been reported as the most frequent neurological disorder found in patients with human immunodeficiency viral infection (Keswani et al. 2002; Morgello et al. 2004; McArthur et al. 2005; Ellis et al. 2010). Damage of the nerve fibers caused by the virus first appears in the lower extremities, impedes sensory capacity and mobility and is often painful (Harrison and Smith 2011; Cherry et al. 2012). Impairment in quality of life is often underestimated and the adverse change in lifestyle often culminates in psychological impact including depression (Griswold et al. 2005; Robinson-Papp et al. 2010; Shaikh et al. 2013; Phillips et al. 2014; Pillay et al. 2018).

Sadly, PN was not only shown to be associated with HIV infection itself but also in many cases triggered by the respective treatment with antiretroviral agents (Keswani et al. 2002; Cornblath and Hoke 2006; Cherry et al. 2012; Schutz and Robinson-Papp 2013; Margolis et al. 2014). The introduction of antiretroviral therapy (ART) has led to significant worldwide reduction of morbidity and mortality caused by HIV/AIDS (Scanlon and Vreeman 2013; UNAIDS 2016). Recommended as initial treatment are nucleoside reverse-transcriptase inhibitors (NRTIs), followed by integrase strand transfer inhibitors, and boosted by protease inhibitors or combinations thereof (Kamerman et al. 2015; Gunthard et al. 2016). Growing evidence of occurring unwanted side complications through exposure to these medications has led to increasing use of safer ART in many countries (World Health Organisation 2015; Ngassa Mbenda et al. 2017).

Still, PN remains prevalent as the most common neurological disorder among people living with HIV/AIDS (PLHIV). A number of studies show that HIV-PN is experienced by 30–60% of PLHIV, surely counting up to several millions of affected worldwide (Ferrari et al. 2006; Evans et al. 2008; Ghosh et al. 2012; Kamerman et al. 2012; Cherry et al. 2016). Albeit rates of sensory neuropathies among HIV patients were reported particularly high in African cohorts (Boulle et al. 2007; Forna et al. 2007; Hawkins et al. 2007; Hoffmann et al. 2008; Mehta et al. 2010), PN is both underdiagnosed and undertreated in low- and middle-income countries. Owing to the fact that sub-Saharan Africa has the highest population of affected PLHIV, scarce PN prevalence data from the African continent indicates a large underrepresentation of research including these population groups (Kamerman et al. 2012; Arenas-Pinto et al. 2016; Ngassa Mbenda et al. 2017).

This study was performed in the largest tertiary referral hospital in Ghana, West Africa, between 2013 and 2014. To our knowledge, this is the first investigation of PN prevalence among PLHIV in Ghana. From our routine clinical management of HIV-PN patients admitted to the health facility, we had strong presumptions on (i) a relatively high prevalence of PN developing in PLHIV, (ii) an association with the medication given in accordance with the guidelines of the National AIDS Control Programme regulations, and (iii) we had assumptions, but no information, on health determinants being associated with PN onset among the patients attending. Once a diseased person develops the condition of a sensory disorder, clinicians face challenges to manage the case, and prevention of PN would both decrease disability and lower the costs of local health care. Therefore, in this study, we (i) sought to quantitatively determine PN prevalence among PLHIV at the referral health facility in Ghana, (ii) determine associations of PN development with demographic and health determinants, and (iii) identify and confirm potential risk factors, including ART, in a West African setting.

For this, we studied parameters in a cohort of PLHIV developing PN including demographic parameters, anthropogenic variables, behavioral aspects, and clinical findings. PN manifestation was assessed through quantitative measurement applying the biothesiometer instrument, and blood samples were collected from the study subjects for downstream analysis of laboratory parameters.

## Methods

### Study design

For this work, a hospital-based cross-sectional study was conducted at the Korle-Bu Teaching Hospital (KBTH) in Accra, the largest tertiary hospital in Ghana, with a capacity of 2000 beds and an outpatient department attendance of over 350,000 in 2016. Patients were recruited from the adult HIV outpatient clinic at the infectious disease unit attending about 19,000 PLHIV at the time of the study, 7000 of which being on ART. Out of the pool of regular attendants to the outpatient clinic, the study population was recruited. Patients were informed on objectives and goal of the study, and informed consent was sought for each participant in written form for involvement in the study. Inclusion criteria encompassed adults with more than 18 years of age who regularly attend the infectious disease unit and were diagnosed with HIV-AIDS. Diagnosis of HIV was made at the infectious unit by antibody detection on blood samples followed by laboratory confirmation using the OraQuick test according to the Ghana National AIDS Control Programme guidelines. A participant was declared positive for HIV if both tests were positive. The sample size was estimated using StatCalc (Epi Info 2002) computer software considering the following parameters: (i) population size of Greater Accra~2,500,000, (ii) expected a frequency of HIV-PN = 5%, and (iii) worst acceptable result = 1%. StatCalc yielded a sample size of 114 at a confidence level of 95%. Assuming 20% (i.e., 23) non-participation/non-response, the total sample size then would be 114 + 23 = 137. An extra number of 163 patients were added to make a total of at least 300. Participants were recruited by systematic random sampling with five patients per day inviting the first patient of each full hour. None of the patients which were sought for enrollment declined consent. Altogether, 525 HIV-positive patients were recruited to participate in the study between 2013 and 2014.

### Collection of socio-demographic and clinical data

Following informed written consent, a questionnaire was administered by a trained research assistant to the participant in order to systematically obtain the following information: sex, age, level of education, employment status, and alcohol and tobacco consumption. Consequently, participants underwent clinical examination to ascertain information on diabetes, hypertension, and any history thereof. Anthropogenic measurements were taken to document height, weight, hip girth, and waist girth.

### Biothesiometric examination

As part of the physical examination, the use of a biothesiometer was applied in order to diagnose PN as objectively as possible. A handheld biothesiometer (BioMedical Instruments, Newbury, OH, USA) which vibrates at 100 Hz when operating on 50 Hz mains was applied to measure the vibration perception threshold (VPT) to determine neurological deficits and for which we expected both specificity and sensitivity of above 70% (Yajnik et al. 2012; Gill et al. 2014). Impaired VPT was measured in three recordings on the plantar aspect of the distal phalanx of the big toe. Each participant was seated in a comfortable and relaxed position and was made to feel the vibration sense of the biothesiometer and to report the initial feel by saying “yes”. The device was held steadily over the testing site such that the weight of the vibrator exerts a standard pressure. The amplitude of the vibration was gradually increased from zero to a maximum of 50 V. For participants who were unable to feel any vibration even at the maximum amplitude, the value of 50 V was recorded. In order to circumvent usage variation and interpretation of the biothesiometer by different physicians, training on the device was given prior to the onset of the investigation. A VPT value of > 9 V was defined as the manifestation of PN.

### Laboratory analysis

Fasting blood samples were collected from each participant for further laboratory analysis at a local commercial laboratory facility. Blood was tested for lipoprotein, lactate, hemoglobin, and fasting glucose. CD4 leukocytes were counted, as were neutrophils.

### Data entry

Documentation of the study included the study protocol, the investigators’ log sheet, the patients’ log sheet, the informed consent, and the questionnaire. From the paper-based and filled case report form, data were entered into the Epi Info software and combined with data retrieved from laboratory analyses. Captured data per participant was cleaned and data transfer periodically checked by trained personnel, followed by application of blinding methodology. Subsequently, data entry of 53 (10%) randomly selected subjects of the 525 patient documentations was reproduced by a person that was neither involved in the study nor in data entry. Among those, only two parameters of the whole raw data set differed which had no impact on the analysis. This random test confirmed the reliability of the entire data set for further analysis.

### Statistical analysis

Continuous factors were summarized as means and standard deviations and categorical factors as counts and proportions. Categorized data were additionally analyzed using underlying continuous values in order not to reduce statistical power.

Medians and interquartile ranges were also calculated but are not presented. In order to examine the association between potential risk factors of PN, firstly, unadjusted comparisons were calculated. Unadjusted comparisons were done using a Chi-square test or Fisher’s exact test if the parameter was categorical, and the Mann-Whitney *U* test if the parameter was continuous. In a second step, multivariable logistic regressions were performed to determine the associations between PN as a binary variable and potential risk factors. Factors known from the literature to have an association with PN and factors with an unadjusted association at *p* < 0.20 were included in the initial multivariable model. The risk factors included in the initial model were being on ART, change of ART medication, patient’s sex and age, education, height and height to weight ratio, hip and waist girth, lactate, chromium concentration, neutrophils and lymphocytes, and CD4 cell counts. The model was reduced using backwards selection with a threshold alpha of 0.25. The statistical software SAS version 9.4 (2002–2012, SAS Institute Inc., Cary, NC, USA) was used to perform the analysis. Statistical significance was set to an alpha level of 0.05. The dataset was not complete for all parameters throughout the 525 subjects, and therefore, almost one-third of the subjects could unfortunately not be included in the multivariable analyses. In a sensitivity analysis, in order to examine the effect of the absence of those respondents, inverse probability weights were calculated and included in the logistic regression, and results were compared to those from the main analysis.

## Results

A total of 525 HIV-positive study participants were recruited for the study. Four hundred and thirty-seven (84.4%) were female; 81 (16.6%) were male. The mean age was 33.6 ± 4.9 with the minimum and maximum age of 19 and 40, respectively. Results are outlined in Table 1 including education and employment status, behavioral aspects (consumption of alcohol and tobacco), and body measurements (height, weight, and hip and waist girth). The mean BMI was calculated as 23.9 ± 4.9 and ranged from 11.3 to 44.6 (not shown). Table 1 also lists laboratory measures encompassing CD4 cell counts, lipoprotein, lactate, hemoglobin, and fasting glucose values.

**Table 1:** Socio-demographic and clinical characteristics: distribution of patients with HIV at a tertiary hospital in Ghana, stratified by receiving ART or not

Characteristic	Total (*n* = 525) *n* (%)	On ART (*n* = 314) *n* (%)	Not on ART (*n* = 211) *n* (%)	*p* value*
Sex				< 0.001
Male	81 (15.6)	34 (11.0)	47 (22.6)	
Female	437 (84.4)	276 (89.0)	161 (77.4)	
Age **(**mean ± SD)	33.6 ± 4.9	34.6 ± 4.3	32.0 ± 5.4	< 0.001
Education				0.019
None	88 (17.1)	61 (19.7)	27 (13.1)	
≤ 9 years	253 (49.0)	154 (49.7)	99 (48.1)	
9–12 years	123 (23.8)	72 (23.2)	51 (24.8)	
> 12 years	50 (9.7)	21 (6.8)	29 (14.1)	
Others	2 (0.4)	2 (0.7)	0 (0)	
Employment status				0.302
Unemployed	104 (20.5)	62 (20.5)	42 (20.6)	
Part-time	22 (4.3)	14 (4.6)	8 (3.9)	
Full-time employment	365 (72.0)	221 (72.9)	144 (70.6)	
Retired and not working	0 (0)	0 (0)	0 (0)	
Retired but working	1(0.20)	0 (0)	1 (0.5)	
Lost employment when status was discovered	1 (0.20)	1 (0.33)	0 (0)	
Too weak to continue working	14 (2.8)	5 (1.7)	9 (4.4)	
Alcohol consumption				< 0.001
Yes, currently	52 (10.0)	14 (4.5)	38 (18.2)	
No	469 (90.0)	298 (95.5)	171 (81.8)	
Smoking status				0.209
Current smoker	10 (1.9)	4 (1.3)	6 (2.9)	
Non/ex-smoker	512 (98.1)	309 (98.7)	203 (97.1)	
Height (cm, mean ± SD)	162.7 ± 8.0	161.8 ± 7.9	163.9 ± 8.1	0.002
Weight (kg, mean ± SD)	63.5 ± 13.4	64.1 ± 13.0	62.6 ± 14.0	0.172
Hip girth (cm, mean ± SD)	79.1 ± 30.8	89.8 ± 25.3	63.7 ± 31.5	< 0.001
Waist girth (cm, mean ± SD)	66.8 ± 27.0	73.3 ± 22.0	53.4 ± 27.7	< 0.001
Peripheral neuropathy				0.201
Yes	93 (17.7)	50 (15.9)	43 (20.4)	
No	432 (82.3)	264 (84.1)	168 (79.6)	
CD4 cell count **(**mean ± SD)	448.2 ± 271.8	493.1 ± 255.7	385 ± 281.8	< 0.001
Low-density lipoprotein (μmol/l, mean ± SD)	3.1 ± 0.9	3.3 ± 0.9	2.9 ± 0.9	< 0.001
High-density lipoprotein (μmol/l, mean ± SD)	1.3 ± 0.4	1.5 ± 0.4	1.1 ± 0.3	< 0.001
Lactate (mmol/l, mean ± SD)	1.8 ± 0.4	1.7 ± 0.4	1.9 ± 0.3	< 0.001
Hemoglobin (g/dl, mean ± SD)	11.9 ± 1.7	12.0 ± 1.6	11.8 ± 1.8	0.049
Fasting glucose (mmol/l, mean ± SD)	4.7 ± 0.6	4.7 ± 0.7	4.7 ± 0.5	0.800

*Chi-square or Fisher’s exact test was used if characteristic was categorical and Mann-Whitney *U* test if continuous

*ART*, antiretroviral therapy; *SD*, standard deviation

Peripheral neuropathy = vibration perception threshold > 9 V, base of first toe (left and right; average of three measurements)

Of the 525 respondents, 314 (59.8%) were on ART. Out of these 314 patients on ART, 281 could give information on the duration on ART, ranging between 1 to 248 months and with the mean of 39.9 ± 30.4 months. Table 2 shows the ART regimen groups administered to the enrolled patients. The prevalence of PN among study participants was 17.7%—93 of the 525 subjects were diagnosed with the neurological disorder above the VPT using the biothesiometer. Of the 93 respondents with diagnosed PN, 50 were on AR therapy and 43 were not.

**Table 2:** Descriptive statistics of the administered ART regimen for all subjects, stratified by PN status

Abbreviation	ART regimen	PN status	All patients (*n* = 525)
			*n* (%)
NRTI	Nucleoside reverse transcriptase inhibitors	No PN	218 (41.52)
		PN	307 (58.47)
NNRTI	Non-nucleoside reverse transcriptase inhibitors	No PN	235 (44.76)
		PN	290 (55.23)
PI	Protease inhibitors	No PN	512 (97.52)
		PN	13 (2.476)

PN (peripheral neuropathy) = vibration perception threshold > 9 V, base of first toe (left and right; average of three measurements)

Table 3 outlines, in an unadjusted manner, potential association between PN and anthropometric, socioeconomic, and clinical characteristics of HIV-positive respondents. Factors of statistical significance (*p* < 0.05) towards development of PN include (i) body height, (ii) waist girth, (iii) hip girth, (iv) neutrophil counts, (v) lymphocyte counts, and (vi) chromium level.

**Table 3:** Unadjusted associations between peripheral neuropathy (PN) and predicting characteristics in HIV patients (*n* = 525)

Characteristic	PN (*n* = 93) *n* (%)	No neuropathy (*n* = 432) *n* (%)	*p* value*
Sex			0.433
Male	17 (18.3)	64 (15.1)	
Female	78 (81.7)	361 (84.9)	
Age (mean ± SD)	34.1 ± 4.8	33.5 ± 5.0	0.284
Education			0.147
None	13 (14.0)	75 (17.7)	
≤ 9 years	55 (59.1)	198 (46.8)	
9–12 years	21 (22.6)	102 (24.1)	
> 12 years	4 (4.3)	46 (10.9)	
Others	0 (0.0)	2 (0.5)	
Employment status			0.392
Unemployed	15 (16.3)	89 (21.4)	
Part-time	4 (4.3)	18 (4.3)	
Full-time employment	69 (75.0)	296 (71.3)	
Retired and not working	0 (0.0)	0 (0.0)	
Retired but working	1 (1.1)	0 (0.0)	
Lost employment when status was discovered	0 (0.0)	1 (0.24)	
Too weak to continue working	3 (3.3)	11 (2.7)	
Alcohol consumption			0.450
Yes, currently	7 (7.5)	45 (10.5)	
No	86 (92.5)	383 (89.5)	
Smoking status			0.222
Current smoker	0.0	10 (2.3)	
Non/ex-smoker	93 (100.0)	419 (97.7)	
Height (cm, mean ± SD)	1.7 ± 0.8	1.6 ± 0.8	< 0.001
Weight (kg, mean ± SD)	65.3 ± 14.5	63.1 ± 13.2	0.232
Waist girth (cm, mean ± SD)	57.1 ± 28.0	69.0 ± 26.3	0.002
Hip girth (cm, mean ± SD)	67.0 ± 31.7	81.7 ± 30.0	< 0.001
ART			
Yes	50 (53.8)	264 (61.1)	0.201
No	43 (46.2)	168 (38.9)	
CD4 cell count (mean ± SD)	407.3 ± 247.9	457.3 ± 276.4	0.236
Low-density lipoprotein (μmol/l, mean ± SD)	3.1 ± 0.9	3.2 ± 0.9	0.484
High-density lipoprotein (μmol/l, mean ± SD)	1.3 ± 0.5	1.3 ± 0.4	0.182
Lactate (mmol/l, mean ± SD)	1.9 ± 0.3	1.8 ± 0.4	0.072
Hemoglobin (g/dl, mean ± SD)	11.8 ± 1.7	11.9 ± 1.7	0.879
Neutrophils (cells/μl, mean ± SD)	41.1 ± 12.6	38.1 ± 11.7	0.033
Lymphocytes (cells/μl, mean ± SD)	48.5 ± 12.4	51.8 ± 11.9	0.020
Fasting glucose (mmol/l, mean ± SD)	4.7 ± 0.5	4.7 ± 0.6	0.795
Chromium (units, mean ± SD)	68.1 ± 23.5	62.6 ± 17.7	0.037

*Chi-square or Fisher’s exact test was used if characteristic was categorical and Mann-Whitney *U* test if continuous

*ART*, antiretroviral therapy; *SD*, standard deviation

PN (peripheral neuropathy) = vibration perception threshold > 9 V, base of first toe (left and right; average of three measurements)

Multivariable analyses of PN with ART and other risk factors are shown in Table 4. Re-analyses of the data on height and age using continuous variables give the same results.

**Table 4:** Multivariable* association of PN with ART and other risk factors (*n* = 333)

Risk factor	Value/level	Adjusted odd ratio	95% CI for OR	*p* value
Intercept				0.010
On ART medication	Yes vs. no, at CD4 = 600 cells	2.17	0.93 to 5.05	0.066
Interaction of on ART and CD4 cell count				0.007
CD4 cell count	10-unit increase for on ART	1.01	1.00 to 1.03	0.033
	10-unit increase for not on ART	0.98	0.97 to 1.00	
Lactate	> 2.2 vs. ≤ 2.2 mmol/l	2.49	0.92 to 6.72	0.073
Age group	> 34 years vs. ≤ 34 years	1.58	0.88 to 2.83	0.124
Education group (years of school)	> 9 years vs. ≤ 9 years	0.49	0.26 to 0.92	0.027
Patient’s height (quartiles)^§^	> 1.66 vs. ≤ 1.57 m	5.74	2.11 to 15.62	< 0.001
	> 1.62 to 1.66 vs. ≤ 1.57 m	4.89	1.69 to 13.66	0.003
	> 1.57 to 1.62 vs. ≤ 1.57 m	3.84	1.38 to 10.66	0.010
Average waist girth	1-cm increase	1.04	0.98 to 1.09	0.159
Average hip girth	1-cm increase	0.96	0.92 to 1.00	0.054

*A logistic regression model was used. The original model included in addition the factors: change of ART medication, patient’s sex, alcohol intake, height to weight ratio, and neutrophils and lymphocyte counts. The model was reduced using backwards selection excluding factors with a *p* value > 0.25

^§^*p* = 0.007 for the overall effect of height

*ART*, antiretroviral therapy

PN (peripheral neuropathy) determined with vibration perception threshold > 9 V (base of first toe, left and right; average of three measurements)

**Fig. 1:**
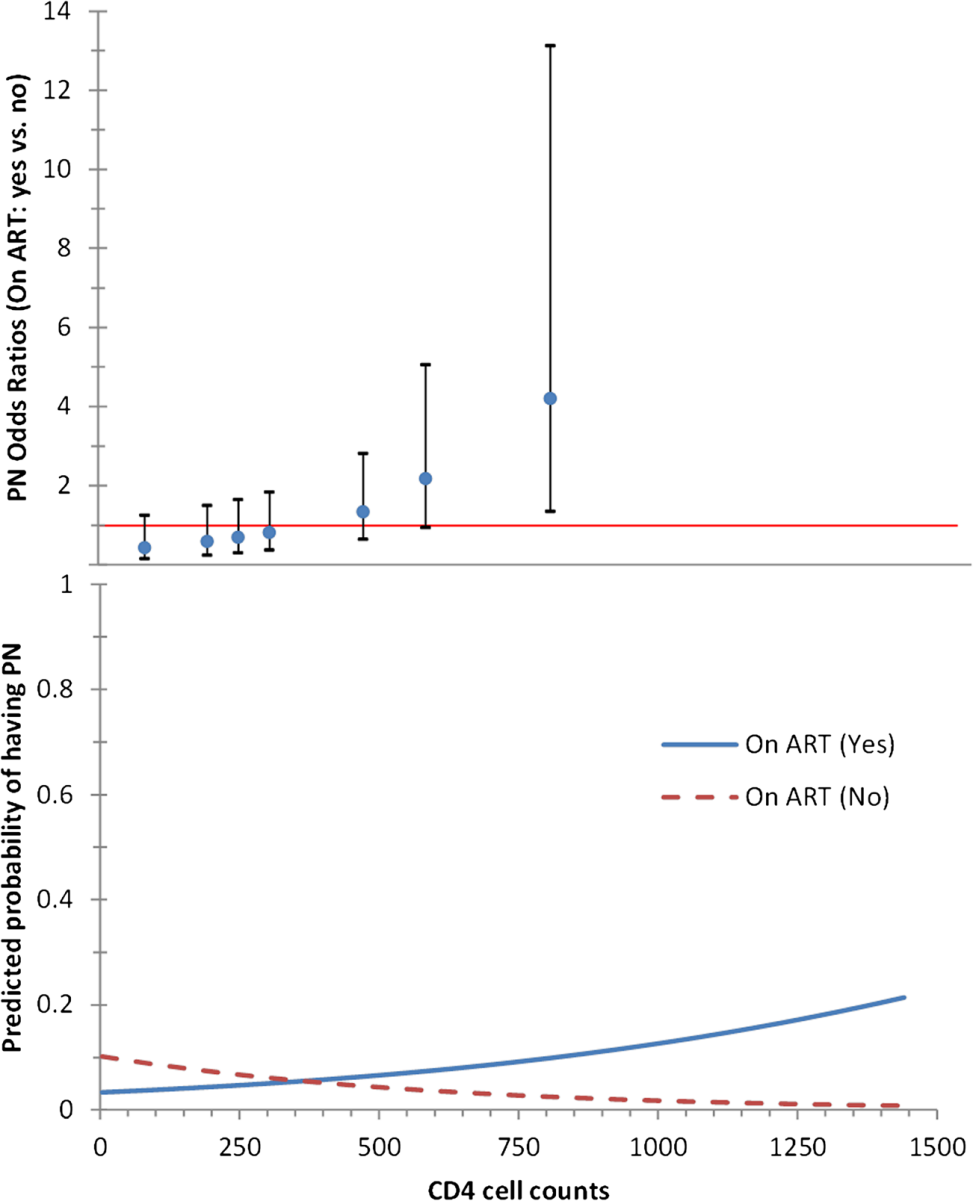
PN odd ratios (OR) for patients on ART (yes vs. no) with calculated predicted probability of having PN in relation to CD4 cell counts (adjusted for relevant factors as shown in Table 4). Top panel: OR of diagnosed PN in patients on ART at different CD4 cell counts (knots show the OR; whiskers the 95% CIs). Bottom panel: effect of the interaction between being on ART and CD4 cell counts on the PN-predicted probability (calculated at the mean values of 65.05 for waist girth, 77.04 for hip girth, lactate < 2.2 mmol/l, a height < 1.57 m, and education of < 9 years)

Surprisingly, the study found a significant association between being on ART and CD4 cell counts (*p* = 0.007). In the presence of an interaction, both antiretroviral (AR) medication and CD4 cells had an effect on PN development (*p* = 0.066 and *p* = 0.033, respectively). For patients on ART, an increase of 10 CD4 cell count units increased their chance of developing PN by 1% (adjusted odd ratio (aOR) = 1.01; 95% CI 1.00 to 1.03). In contrast, for patients not on ART, such increase of 10 units in CD4 cell counts decreased their chance of developing PN by 2% (aOR = 0.98; 95% CI 0.97 to 1.00) (Table 4). Figure 1 visualizes this interaction between developing PN and being on ART. The aOR are as follows: 0.69 (95% CI 0.29–1.64) at a CD4 cell count of 250; 1.33 (95% CI 0.63–2.81) at a CD4 cell count of 450; and 2.17 (95% CI 0.93–5.05) at a CD4 cell count of 600. Hence, the probability of being diagnosed with PN—when being on ART or not—in relation to CD4 cell counts could be estimated (Fig. 1).

Statistical significance towards developing PN was also found to be associated with an increase in lactate levels (*p* = 0.073) and with age over 34 years (*p* = 0.124). Instead, patients with > 9 years of education have a lower risk of suffering PN (*p* = 0.027). With a statistically significant overall effect of body height on PN (*p* = 0.007), study participants between 1.57–1.62 m of height had 3.84 times the aOR of developing PN compared to those with heights ≤ 1.57 cm (95%CI 1.38 to 10.66). This aOR increases to 4.89 and 5.74 times the aOR of subjects between 1.62–1.66 m (95% CI 1.69 to 13.66) and > 1.66 m (95% CI 2.11 to 15.62), respectively.

No statistical significance among PLHIV with PN was found in sex, age, education and employment status, alcohol and tobacco consumption, or weight. Sensitivity analysis using inverse probability weighting to examine the effect in patient datasets with missing information did not show different associations than the presented model (see Table S1 in the supplementary material).

## Discussion

The introduction of antiretroviral medication has drastically reduced morbidity and mortality. Although management of HIV/AIDS cases through the use of ART has improved quality of life and a longer survival, chronic co-morbidities have emerged. Also, increased life expectancy naturally made the HIV-infected population grow older (Scanlon and Vreeman 2013; World Health Organisation 2015; UNAIDS 2016). Hence, the continued high prevalence of sensory neuropathies continues to affect PLHIV and requires clinical and health care attention (Ellis et al. 2010; Schutz and Robinson-Papp 2013).

The present investigation including 525 patients is the largest study on PN so far in Ghana and, to our knowledge, in West Africa, carrying the highest burden of HIV infection worldwide. In an earlier study, PN prevalence was revealed in type 2 diabetes patients in the same setting (Yeboah et al. 2016). The prevalence of PN in this study’s subset of individuals admitted to the largest tertiary referral hospital in Ghana in 2013/2014 and infected with HIV/AIDS was 17.7%. This number somewhat ranges within the ratios found in other studies performed in sub-Sahara Africa. Such studies reported prevalence of 6% and 57% in South Africa, respectively (Boulle et al. 2007; Wadley et al. 2011), 13% in Malawi (Beadles et al. 2009), 20.7% and 36% in Kenya, respectively (Hawkins et al. 2007; Mehta et al. 2010), 36% in Uganda (Forna et al. 2007), 24% in Uganda/Zimbabwe (Kiwuwa-Muyingo et al. 2014), 59% in Rwanda (Tumusiime et al. 2014a), and 28% in Cameroon (Luma et al. 2012). A recent study by Benevides et al. 2017 in southern Brazil unveiled 31.3% of PN prevalence in PLHIV. In some settings, variations in the methodology to diagnose PN even seem to cause over-reporting of PN (Tumusiime et al. 2014b).

These obvious differences may originate in the differing nature of the performed studies: (i) the sample sizes differ considerably, with our study representing a respectable patient number, (ii) the inclusion criteria applied, resulting in varying average age of the study subjects, (iii) varying progression stages of the underlying HIV/AIDS infection of the study subjects, (iv) altering diagnostic approaches and procedures applied, (v) the ratio of subjects being on antiretroviral medication, and (vi) the different type of ART regimen administered to the study cohorts.

With regard to the latter, in many studies, patients were enrolled whilst on NRTIs or protease inhibitors as a first-line antiretroviral medication. It was repeatedly reported that these drug groups have severe neurotoxic impact, above all stavudine, but also didanosine and zalcitabine (Forna et al. 2007; Hoffmann et al. 2008; Ances et al. 2009; Maritz et al. 2010; Evans et al. 2011; Wadley et al. 2011; Kamerman et al. 2012; Chen et al. 2013; Kiwuwa-Muyingo et al. 2014; World Health Organisation 2015). In Ghana, since inception of the study, the first-line treatment is a combination of two NRTIs and one non-NRTI, followed by a second-line treatment also including a protease inhibitor.

Increased CD4 counts may account for either immunocompetence or viral control due to treatment. Studies on subject under NRTI treatment regimen usually did show low CD4 levels associated with PN whereas later studies, omitting NRTIs and protease inhibitors, tend to show no association between CD4 counts and sensory neuropathies (Evans et al. 2011; Benevides et al. 2017). The underlying mechanisms are unclear and require additional research. Our unusual finding of increased CD4 lymphocytes associated with PN only in HIV infected on ART but not in non-treated patients remains an undecided observation and may be interpreted such that the treatment reduces viral load but continues to negatively affect patients’ nervous conditions.

It was reported earlier that women are more affected by development of PN (Collins et al. 2009; Nwabueze et al. 2012; Gomez-Olive et al. 2013; Kimanga et al. 2014; Fagbamigbe et al. 2016) which is confirmed in this study. Nevertheless, this does not necessarily mean that women are more susceptible to development of PN. Women’s more prudent health care–seeking behavior when confronted with illness, accounting for a higher detection rate in women, is a likely explanation for this finding.

Accordingly, higher education usually leads to better awareness of both a healthy lifestyle and handling of illness including compliance to medication, and this coincides with this study’s findings of a lower risk of PN development in patients with higher education status. Surely, this association supports the aspirations of the sustainable development goals no. 1 “no poverty,” no. 4 “quality education,” and no. 10 “reduced inequalities” also in this particular diseases.

Our study revealed that increasing height was independently associated with the risk of developing of PN, reconfirming the findings of other studies. This is probably simply owing to the fact that increased nerve length, and thus a larger axon surface area, may be of greater risk for neurological damage (Cheng et al. 2006; Chen et al. 2013; Kote et al. 2013; Luma et al. 2018).

In contrast to the recently published linkage between use of tobacco and PN development in Brazil (Benevides et al. 2017), our data does not confirm such association.

Age as a risk factor for PN, however, was reported many times before and proved to be so in HIV patients above 34 years of our cohort as well (Evans et al. 2011; Wadley et al. 2011; Nwabueze et al. 2012; Chen et al. 2013; Kiwuwa-Muyingo et al. 2014; Tumusiime et al. 2014a, b). As possible explanation, on the one hand, aging increases vulnerability towards neuronal toxicity, on the other hand is the HIV population’s median age rapidly increasing due to continuously improved management of infected persons in Sub-Sahara Africa, and so is the prevalence of PN. Therefore, these results encourage health systems to routinely screen PLHIV for early onset PN of age > 30 years.

The here determined association between PN in HIV patients with levels of chromium (Head 2006; Kallianpur et al. 2006; Afridi et al. 2014) and lactate (Miller et al. 2002; Brew et al. 2003) as well as elevated counts of neutrophils as compared to CD4 lymphocytes (Ford et al. 2015; Means et al. 2016) indicates that blood testing including these parameters may be a good indicator when assessing early onset development of PN.

Meanwhile, genetic studies made assumptions on single-nucleotide polymorphisms, i.e., in gene CAMKK2, on position rs28445017, or in MTND2*LHON-4917G but possibly also others, being associated with development of PN but this needs further clarification (Canter et al. 2008; Wadley et al. 2015; Goullee et al. 2016; Hendry et al. 2016; Gaff et al. 2018).

Surely, our study had limitations. The cross-sectional design of our study did not allow for detecting PN prior to ART initiation nor for longitudinal comparison between antiretroviral naïve PLHIV and those on ART. Vibratory sense deficits may be caused also by other factors which may have confounding effects to the results. Only a few of our study participants were on protease inhibitor–based regimen so that the influence of this drug class may be undervalued. Difficult to quantify during the interview and thus equally underestimated is substance consumption (alcohol and tobacco). Since patients in the geographic setting usually seek medical attention late, also the information on the mean duration of HIV infection may be inaccurate. And finally, confounding effects with i.e., cell folate deficiency, thyroid dysfunction, hepatitis B and C infection, paraproteinemia, and any other potentially neurotoxic drugs cannot be entirely ruled out in the study approach.

During the investigational period, we felt vindicated by our approach of using the biothesiometer as a quantitative method to determine PN for comparative diagnosis. However, during the analyses phase, we sensed that complementing method(s) could have underpinned the case definition and hence the identified prevalence. We assumed both specificity and sensitivity of above 70%; however, reports reveal a wider range (England et al. 2005; Gill et al. 2014). In our view, the biothesiometer should be accompanied by the observatory clinical assessment of PN, the application of the monofilament methodology, or even the Michigan neuropathy screening instrument (MNSI) which combines both symptoms and clinical examinations and had been validated by earlier studies to be accurate and reliable for screening PN (England et al. 2005; Yajnik et al. 2012; Gill et al. 2014; Woldeamanuel et al. 2016).

## Conclusions

Derived from the observation during our routine clinical management of HIV-PN patients admitted to the Korle-Bu Teaching Hospital in Accra, Ghana, our assumption of a relatively high prevalence of PLHIV developing PN was in a way confirmed albeit the situation is less severe than presumed. Possibly, the phasing out of the NRTIs didanosine, stavudine, and zalcitabine in ART regulations by the Division of Public Health under Ghana Health Service in 2012/2013 fortunately reduced incidences of sensory neuropathies. Our results endorse inclusion of safer ART regimens, known to have less severe secondary effects, in respective national antiretroviral regulations and continuation of developing and testing better tolerable drugs against HIV infections.

The unveiled associations of PN with demographic, anthropometric, behavioral, and laboratory parameters confirmed earlier findings, above all body height and age from 34 years on as risk factors.

Our CD4 cell monitoring results, showing PN development associated with CD4 lymphocyte increase only triggered by ART but not in non-treated PLHIV, may hint towards possible antiretroviral secondary effects. Such would need to be particularly looked at in the sub-Saharan African setting which bears among the highest HIV/AIDS disease burden.

The biothesiometer is a reliable tool for quantitative PN diagnosis albeit the need for more robust and comparable assessment remains obvious.

The prevalence of still 17.7% of PN among PLHIV indicates that there is still a need for further reduction in morbidity of neuronal damage even in the post-stavudine era. Inacceptable complications, adding to the disability of the affected, can be counteracted with surveillance, early identification, and case management, requiring continued attention by the health system. Early health care seeking should be encouraged for both men and women, and PLHIV from the age of 30 on should be routinely screened for PN.

## Electronic supplementary material

ESM 1 (DOCX 19 kb)
