# Supplementary material for: Peripheral neuropathy in patients with human immunodeficiency viral infection at a tertiary hospital in Ghana
Source: J Neurovirol. 2019 Apr 26;25(4):464–74. doi: 10.1007/s13365-019-00743-0 (PMC6746675; doi:10.1007/s13365-019-00743-0)
Supplement: Supplementary file 1 — (DOCX 19 kb) [file 13365_2019_743_MOESM1_ESM.docx]

Table 1S: Multivariable* association of peripheral neuropathy and ART and other risk factors (using inverse probability weights)

| Risk Factor | Value / level | Odds Ratio | 95% CI for OR | p-value |
| --- | --- | --- | --- | --- |
|  |  |  |  |  |
| Intercept |  |  |  | 0.010 |
|  |  |  |  |  |
| On ART medication | Yes vs. No, at CD4=600 cells | 2.19 | 0.88 to 5.43 | 0.060 |
|  |  |  |  |  |
| Interaction of on ART and CD4 cell count |  |  |  | 0.007 |
|  |  |  |  |  |
| CD4 cell count | 10 unit increase for on ART | 1.02 | 1.00 to 1.03 | 0.041 |
|  | 10 unit increase for not on ART | 0.98 | 0.96 to 1.00 |  |
|  |  |  |  |  |
| Lactate | >2.2 Vs. ≤2.2 mmol/l | 2.42 | 0.88 to 6.66 | 0.087 |
|  |  |  |  |  |
| Age group | >34 years Vs. ≤34 years | 1.59 | 0.85 to 2.89 | 0.151 |
|  |  |  |  |  |
| Education group (years of school) | >9 years Vs. ≤9 years | 0.49 | 0.25 to 0.95 | 0.035 |
|  |  |  |  |  |
| Patient’s height (quartiles) | >1.66 Vs. ≤1.57m | 5.74 | 2.10 to 16.03 | <0.001 |
|  | >1.62 to 1.66 Vs. ≤1.57m | 4.72 | 1.63 to 13.68 | 0.004 |
|  | >1.57 to 1.62 Vs. ≤1.57m | 3.77 | 1.34 to 10.64 | 0.012 |
|  |  |  |  |  |
| Average waist girth | 1 cm increase | 1.04 | 0.98 to 1.09 | 0.174 |
|  |  |  |  |  |
| Average hip girth | 1 cm increase | 0.96 | 0.92 to 1.00 | 0.069 |

* A logistic regression model was used. The original model included in addition the factors: change of ART medication, patient’s sex, alcohol intake, height to weight ratio, chromium, neutrophils, and lymphocyte concentration. The model was reduced using backwards selection excluding factors with a p-value>0.25. An inverse probability weight in function of On ART, change ART medication, sex, age, education and height was used to estimate probable effects if all subjects could have been included in the model

ART (Antiretroviral therapy)

PN (Peripheral neuropathy) determined with vibration perception threshold >9V (base of first toe, left and right; average of three measurements)
